# Management of Sickle Cell Disease Pain among Adolescent and Pediatric Patients

**DOI:** 10.3390/brainsci9080182

**Published:** 2019-07-30

**Authors:** Samar Abdo, Khawla Q. Nuseir, Ahmad A. Altarifi, Moussa Barqawi, Nehad M. Ayoub, Tareq L. Mukkatash

**Affiliations:** 1 Department of clinical pharmacy, Faculty of Pharmacy, Jordan University of Science and Technology, Irbid 22110, Jordan; 2 Department of Pharmacology, Faculty of Medicine, Jordan University of Science and Technology, Irbid 22110, Jordan; 3 Department of Pediatrics, Princess Rahma Teaching Hospital, Irbid 21110, Jordan

**Keywords:** assessment, APPT, Jordan, healthcare, hospital

## Abstract

Management of sickle cell pain in adolescent and pediatric patients is inadequate, and the employment of proper management guidelines and practices are highly variable among different regions and populations. APPT, the multidimensional adolescent pediatric pain tool, promotes optimal pain management and introduces best practical guidelines for pain management. The goal of this study is to assess pain and pain management among young patients diagnosed with sickle cell disease (SCD) by introducing the APPT as a tool for pain management, and analyze factors contributing to pain management. Information relevant to demographic data, SCD characteristics, APPT assessment, and satisfaction of patients regarding pain management were collected using a structured questionnaire. Results showed that SCD is highly associated with gender (*p* = 0.022), consanguinity (*p* = 0.012), and number of surgeries (*p* = 0.013). Most patients (58.9%) indicated the involvement of more than six body areas affected during pain crisis. Severe pain was described by more than half the patients (55.6%), while moderate pain was reported by 31.1%. Most patients described their pain by sensory, affective, and temporal words. The number of painful areas, pain intensity, and use of descriptive pain words was correlated and interpreted by age, BMI, school absence, and number of surgeries. Results of this study could provide guidance to healthcare providers to improve current practices for SCD pain management in order to improve health outcomes and patients’ satisfaction.

## 1. Introduction

The global burden of hemoglobin disorders cannot be ignored; they contribute to about 3.4% of mortality in children aged less than five years [1,2]. Worldwide survival depends on the economic status of the country in which the person is born; in high-income countries, affected children live longer, suffering mainly from chronic disorders, while in low-income countries, affected children die before the age of five [1,3].

Sickle cell disease (SCD) is a worldwide, common hereditary blood disorder affecting red blood cells [1]. SCD, a group of autosomal recessive genetic disorders depicted by the sickle hemoglobin (HbS) variant, is frequent in most populations of sub-Saharan Africa, the Middle East, India, as well as Western Europe, and the eastern coast of the Americas following migrations of people from affected areas [1,4,5]. In Jordan, SCD was studied mainly in Northern Jordan where the prevalence of sickle cell traits in newborns was found to be 3.17%, while the estimated prevalence was expected to be 4.45% among newborns [6]. 

SCD displays several symptoms and complications including chronic anemia, acute chest syndrome, stroke, renal dysfunction, susceptibility to bacterial infections, and pain crisis [4,7]. Pain crisis is a hallmark of SCD. Pain crisis is the primary reason for hospitalization or visits to emergency departments. Sickle cell pain could be nociceptive, neuropathic, or mixed [8,9]. The pain is characterized by recurrent, sudden, or triggered (by many factors) episodes. Acute severe pain is mainly due to vaso-occlusive crisis (VOC) [9]. It is also associated with inflammation that worsens with recurrent pain episodes, often terminating in serious complications and organ damage such as acute chest syndrome, multi-organ failure, and sudden death [9]. Management of sickle cell associated pain remains unsatisfactory. Current methods and treatment protocols are borrowed from other painful states rather than being developed for sickle cell pain [9]. Opioids and non-opioids are used to treat sickle cell pain; opioids are associated with many side effects with the potential for addiction [10,11]. Non-opioids are not sufficiently effective in severe pain and are also associated with many adverse effects [12]. Blood transfusion and hydroxyurea used in SCD show reduction in VOC pain crisis [2,13].

Pain crisis is a major clinical manifestation of SCD [11]. Proper assessment of pain is the first step toward adequate pain management. Sickle cell pain experiences are multifaceted with features that cannot be effectively assessed by a numeric rating of pain intensity since this assesses a single dimension of pain [14] Since adequate pain assessment in adolescent and pediatric patients poses a challenge, multidimensional pain assessment tools have been developed [15]. The Adolescent Pediatric Pain Tool (APPT) is a systematic method for relating the characteristics of pain to function, and offers physicians more objective measures for determining the effectiveness of pain relief interventions [16]. The APPT had been used and found to be effective for proper assessment and subsequently better management of SCD pain [15,16,17,18,19,20,21,22,23]. The APPT provides valuable information not only about the intensity but rather the location and quality of pain [23]. Even adaptation of features from the APPT used in a self-reporting pain diary have proven useful for pain assessment and management [22]. Nevertheless, the APPT is underutilized in clinical settings, particularly in the Middle East [24].

Pain management at hospitals in Jordan utilizes both opioids and/or non-opioids for management in SCD pain, and to the best of our knowledge, SCD pain management satisfaction among young patients has not been previously evaluated. Therefore, lack of understanding of the characteristics of SCD pain may lead to both over and under treatment of pain. The purpose of this study is to introduce a multidimensional pain assessment tool (APPT) with the hope of optimizing pain management, improving patient’s satisfaction and boost pain relief outcomes. The objective of this study is to examine SCD pain and pain management by assessing sickle cell pain in adolescent and pediatric patients using clinical practical guidelines, and to explore patients’ satisfaction with their current pain managements. 

## 2. Materials and Methods

This was a descriptive, cross-sectional study, conducted at three hospitals in Jordan between October 2016 and February 2017.

### 2.1. Ethical Approval

Approval was obtained from Jordan University of Science and Technology (number 20150327), and from the Ministry of Health Institutional Research Board (IRB).

### 2.2. Participants and Setting

Out of 125 patients approached, 90 patients completed the survey and record taking. Data was collected from three governmental hospitals located at northern and central regions in the country: Princess Rahma (hospital 1), Al Basheer (hospital 2) and Princess Basma (hospital 3) hospitals in Jordan. 

### 2.3. Procedure

The research goals and methods were explained to the patients and their legal guardians. Patients who agreed to participate in the study were handed consent forms to sign. The questionnaire was administered and collected by the researcher in a semi structured (interview) fashion with the patients or their legal guardians. 

### 2.4. Research Tool

After an extensive literature review, the questionnaire was developed and modified by the authors taking into consideration previous work published in literature regarding the same topic. A pilot study was conducted at the beginning of the study (*n* = 15) in order to consider any modifications to the questionnaire before the start of large-scale data collection. The final version of the questionnaire took into consideration the results of the pilot study. The pilot questionnaires were not considered in the final data analysis. The final version of the questionnaire employed for this study was composed of four sections, which included demographics, disease characteristics, pain assessment and management in hospitals, and patient satisfaction regarding pain management.

### 2.5. Demographics

Patients’ sociodemographic information such as gender, age, and school attendance, as well as parents’ blood relation, educational, and financial status were collected via face-to-face interview of the patients or legal guardians. 

### 2.6. Disease Characteristics

Clinical characteristics of SCD were obtained from the hospital patients’ records, such as the type of SCD, duration of disease, blood test, other co-morbidities, disease management at home, medications administered and adverse effects. 

### 2.7. Pain assessment and Management in Hospitals

Patients were asked about frequency of SCD pain, provocative and/or palliative factors during pain episodes, as well as symptoms associated with pain crisis. Pain crisis was evaluated by using the (APPT). Adolescent Pediatric Pain Tool (APPT) is a multidimensional pain instrument for adolescent and pediatric patients that assesses pain status by the location or body areas, pain intensity, and description of pain using words divided into sensory (describe pain they can feel), affective (describe pain related emotions), and temporal (described pain in relation to time) categories.

Pain intensity was measured by numerical scale for patients five years or older, while for the case of younger patients (less than five years) the parents gave their estimates of their children’s pain intensity. This numerical scale was divided into three categories: mild (0–3), moderate (4–6), and severe pain (7–10). In addition, patients were asked about pain management during hospital stay, and the duration of time it took to get pain relief was recorded.

### 2.8. Evaluation of Patient Satisfaction regarding Pain Management

Patients were asked specific questions which were developed to analyze and assess their satisfaction regarding pain management in hospital settings.

### 2.9. Statistical Analysis

Data analysis was performed using SPSS version 20.0 (IBM Corp., Armonk, NY, USA). As the continuous variables were not normally distributed, non-parametric statistical tests were applied. Continuous variables were presented as medians and interquartile ranges (IQRs) expressed by the 25th and 75th percentiles. Categorical variables were presented as the frequency and percentages. To assess associations between categorical variables, Pearson’s χ^2^ test of independence was used. To assess correlations between continuous variables, Spearman’s correlation test was applied. Findings were considered statistically significant when *p* < 0.05. 

## 3. Results

A total of 90 questionnaires were completed; this amounted to a response rate of 72%.

### 3.1. Sample Attributes

The demographic data for patients in this study are summarized in Table 1. The median age for patients was 10 years (IQR, 6–14). Most patients were admitted to Princess Rahma Pediatric Hospital (hospital 1) (63.3%). The majority of patients (83.3%) indicated irregular school attendance and in more than half, parents were first-degree relatives (54.4%). The median number of siblings among the sample was 3 (IQR, 2–5). The median BMI for patients was 15.5 kg/m2 (IQR, 14–19.1). Other demographic information is shown in Table 1.

### 3.2. SCD Characteristics

Details concerning the characteristics of the SCD are summarized in Table 2. Most patients had SCD of the SS Hb subtype (53.3%), and disease follow up was the main reason for hospital admission (90%). The median count for white blood cells (WBC) was 13.4 × 10^3^/mm^3^ (IQR, 9.2–17.7). The median red blood cell (RBC) count and hemoglobin (Hg) concentration were 3.1 × 10^6^/mm^3^ and 8.2 mg/dL, respectively. Most patients presented with high WBC count (63.3%) and low RBC count (84.4%). All patients had low Hg levels and the vast majority had blood transfusions. Respiratory disease was the most common comorbidity reported.

### 3.3. Associations between SCD Characteristics and Patient Demographic and Clinical Characteristics

**Figure 1:**
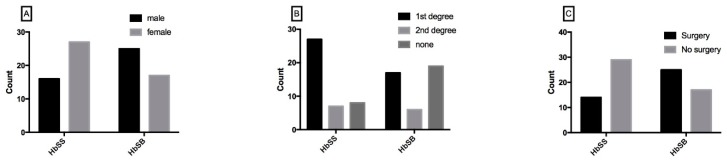
Association between SCD type and patient demographic and clinical characteristics. (**A**) Association between patients’ gender, (**B**) consanguity, and (**C**) surgical history with SCD type.

Chi square analysis showed a significant association between the types of SCD disease and gender. More females had the HbSS genotype (the most common, referred to as sickle cell anemia, results from homozygote inheritance), while males suffered more of the beta thalassemia genotype (HbSβ) (most prevalent in eastern Mediterranean region, results from heterozygote inheritance, χ^2^ = 5.230, *p* = 0.022, Figure 1A). In addition, a significant association between type of and consanguinity was found; first consanguinity marriages resulted in offspring suffering mainly the HbSS genotype, while non-consanguinity among parents resulted in offspring who had more the HbSβ type (χ^2^ = 8.897, *p* = 0.012, Figure 1B). Patients of the HbSβ type had more surgeries than HbSS genotype patients (χ^2^ = 6.195, *p* = 0.013, Figure 1C).

### 3.4. Medication Used at Home

Most patients used analgesics, mainly acetaminophen (75.6%) and non-steroidal anti-inflammatory drugs (NSAIDs) (74.4%), regularly at home for pain management. Almost all patients reported regular use of folic acid (97.8%). Indigestion was the most frequent adverse effect indicated by patients (6.7%). List of medications used and side effects as described by the patients are shown in Table 3.

### 3.5. Causes of Admission and Symptoms associated with Pain Crisis

Pain crisis was the most common cause for hospital admission (93.3%). The median number of pain crisis among the study sample was 5 episodes per patient was (IQR, 3–15). Pain was either provoked (65.6%), or sudden (34.4%). Top provoking factors include extreme cold (81.1%), extra movement or hard work (41.1%), exposure to heat or sun (23.3%), and psychological factors (17.8%). Several symptoms accompanying pain crisis from SCD were reported by patients. The most common were loss of appetite (82.2%), joint pain (81.1%), jaundice (77.8%), fever (80.0%), feeling tired (67.8%), palpitation (56.7%), and shortness of breath (50.0%).

### 3.6. Pain Assessment

Pain characteristics with SCD were evaluated and assessed via APPT. Number of body areas with pain crisis was reported per patient from (0–21) sites, with a median of six sites. About 53 patients (58.9%) had pain crisis in six or more body sites, while 37 patients (41.1%) had pain in less than six sites. Most frequently, pain was in the lower back, chest, hip, abdomen and extremities. Pain intensity was determined by numerical pain scale (NPS); NPS gives objective values of pain as reported by the patients from 0 (no pain) to 10 (most severe pain). All patients reported a degree of pain intensity. The median was 7 (IQR, 4.5–10); 12 patients had mild pain (13.3%), 28 patients reported moderate pain (31.1%), and 50 patients had severe pain (55.6%). Patients described their pain by at least one sensory word per patient such as pins, needles, squeezing, or stabbing; one affective word per patient such as annoying, crying, and screaming; and one temporal word per patient such as slowly, off and on, and continuous as summarized in Table 4.

### 3.7. Correlation Analysis between Demographic and Pain Variables

Bivariate correlation analysis is shown in Table 5. Age and BMI were positively correlated and significantly with pain intensity, the number of painful body areas, and sensory and temporal words. Frequency of school absences were significantly and positively correlated with pain intensity, number of painful areas, and temporal words. A significant and positive correlation was found between pain intensity and all other pain assessment variables (Table 5). A similar trend was found between the number of painful body areas and other pain variables. 

### 3.8. Pain Managements at Hospitals

All patients reported pain relief at the hospital (100%). Medications used at hospital for pain management were either non-opioids for 79 patients (87.8%), or combination of opioids with non-opioids for 11patients (12.2%). The time to pain relief ranged from 10 min to more than 24 h. However, most patients 68 (68, 75.6%) required over 24 h for complete pain relief.

### 3.9. Patients Satisfaction

Most patients (81, 90.0%) were satisfied with pain management at their hospitals, as well as services from with healthcare providers (HCPs). The majority (73, 81.1%) of patients said that medications used were explained to them thoroughly by HCPs.

## 4. Discussion

Until a cure for sickle cell disease is discovered, patients, particularly children will continue to suffer from the pain that accompanies this disease. As healthcare providers, we are all invested in finding ways to assess and treat this pain in an efficient and timely manner. 

In this study, pain crisis was the main reason for hospital admissions (93.3%), in accordance with similar percentages in other published reports [10,23,25,26]. Pain crisis, mainly due to a vaso-occlusive episode, is a main reason for hospital admissions for SCD patients [23,25,26,27,28]. Pain crisis either occurred suddenly (in 34.4% of patients), or was triggered by other factors such as extreme cold, hard work, heat and sun, psychological factors, and deficiency of ingested fluids, also in agreement with previous researches [4,9,24,29]. Furthermore, the patients stated other symptoms with varying degrees among individuals. Symptoms such as loss of appetite (82.2%), joint pain (81.1%), jaundice (77.8%), fever (80%), feeling tired (67.8%), palpitation (56.7%), SOB (50.0%), headache (35.6%), drowsiness (17.8%), nausea, vomiting (14.4%), swelling (11.1%), lack of concentration (4.4%), and enlarged spleen (5.6%). These symptoms might also contribute to the pain and to hospital admissions, and should be considered in pain management decision if possible. Furthermore, symptoms accompanying pain which impact daily function and thus affect quality of life, such as decreased physical activity (38.9%) and irregular school attendance (83.3%), are noteworthy and need to be addressed [18].

This study employed the APPT, a systemic method for describing the characteristics of pain and provides more objective measures, such as number of painful body areas, pain intensity, and description of pain using sensory, affective, and temporal words [17]. The introduction of the APPT to these patients was helpful in assessment and management of pain. Research has shown that employing the APPT provided better awareness regarding pain magnitude regardless of the setting of care [17]. The results showed that 58.9% of patients reported six or more body areas having pain. This study found most common sites were in the lower back, chest, knee, hip, abdomen, and extremities. Similarly APPT at different care settings showed that several patients (33%–68%) reported pain in chest, upper and lower back, and leg [17]. Also chest, abdomen, and lower back were most frequently marked areas (55%) on the APPT by adolescents and children in another study [23]. Pain intensity was moderate for one-third (31.1% of patients) to severe for over half (55.6% of patients) during pain crisis, only a few patients had mild pain (13.3%) comparable to other studies [18,23,26,30]. Pain intensity was positively correlated with number of painful body areas; pain became more intense as number of areas affected by pain increase. Patients used provided sensory, affective, or temporal words to describe their pain [23]. Most frequently used words to describe pain in SCD were pins, needles, squeezing, stabbing, annoying, crying, screaming, off and on, slowly, and continuous. Using different words to describe pain assists in differentiation between nociceptive and neuropathic pain, which is useful for the selection of appropriate pain managements [17,22]. Pain intensity and locations as well as descriptive word for pain were positively correlated with age, BMI, school absences, and surgeries. Age was also a factor in another study; the older children stated more body areas with higher pain, and used more evaluative and temporal words to describe their pain [17]. The association of pain with body mass index was positive in this study, this could be explained, at least partially, by the notion that more pain leads to less movement and thus more weight gain. Studies of SCD adults’ and pediatric patients found out that patients were gaining weight with age, probably as a result of improved treatment and longevity [31,32,33]. Pediatric patients’ length of hospitalization slightly increased with BMI [34]. Also, our findings revealed an increase in pain intensity with an increase in number of painful body sites and number of words used to describe pain by patients.

Medications used for pain management at hospitals are either non-opioids and/or opioids. Suggested guidelines for management of SCD pain include the use of opioid and non-opioid medications in addition to supportive treatment [9,12,21,24]. Opioids are used much less often than non-opioids (12% vs. 87%). Thus, non-opioids were mostly used, in particular acetaminophen and NSAIDs. Misconception and fear of adverse effects, as well as deficiency in knowledge about pain and pain management in general is the main reason for this underuse of opioids. We had shown previously that HCPs lack in knowledge particularly regarding use of opioids and especially for children patients [35]. Results of this study shows that in only few patients’ pain relief occurred within 10 min, while most patients (75.6%) needed over 24 h to achieve complete pain relief. Average length of hospital stay varies from three days [27] to over 10 days [26], which make us wonder about patients claiming total pain relief within 24 h. In addition to the suffering of patients, this requires a longer stay at the hospital and thus extra costs. Furthermore, this causes inconveniences for the family or caretakers of the patient, particularly since most of the patients hail from poor families and come from distant villages and towns. A recent study from Jordan has shown that caregivers, mainly parents, of SCD pediatric patients indeed suffer from many psychological and physical hardships [36]. Parents of these children, particularly the mothers, endure enormous strains and difficulties.

Most patients (90.0%) were satisfied with pain management, which was surprising considering that pain relief took over 24 h for most patients. We might explain this by the good patient phenomenon; the patients feel like saying good things about healthcare providers out of fear or shyness [20]. SCD patients need rapid assessment and pain management within 30 min from admission to the hospital as well as reassessment following analgesic administration, every 15 min until total pain relief. Using APPT as practical guideline for assessment will help improve health care. In addition, pain intensity changes over time, therefore, the APPT assessment should be used regularly to determine the effectiveness of pain relief intervention and modify the doses of pain medications. Moreover, APPT can be used not only for hospitalized patients but also in outpatient clinics, community facilities and at home. The impact of a clinical pharmacy service on the management of patients in a SCD is very important and necessary, as is follow up with the patient and explanation of proper use of medications, analgesic of pain, hydroxyurea dose, folic acid, and regular immunization injections. These practices are associated with improved results, and therefore lead to decrease frequent episode of SCD and increased health care outcome [21].

## 5. Conclusions

In conclusion, pain crisis continues to play an integral part in increasing morbidity and mortality among patients with SCD. Understanding the nature of SCD and pain crisis by health care providers will be instrumental in offering the best managements of pain. Thus, early pain assessment and management can help in reducing morbidity and mortality. The APPT is widely used among pediatric populations with a variety of clinical conditions. Use of the APPT provides a lot of information that aids in understanding of the development of pain over time. In addition, APPT can be used in acute pain for both hospitalized and non-hospitalized adolescent and pediatric patients. APPT not only measures pain intensity, but is also a multidimensional pain-assessment tool for those experiencing complex pain situations. Multidimensional pain-assessment tools promote cooperation between clinicians and patients. Assessment tools should be recommended for all pain situations, and healthcare providers should be encouraged the use these tools. 

## 6. Limitations

- Cross sectional study allowed for one-time only measure of outcome and exposure thus limiting examination of cause and effect.
- Even though the sample size was decent, more participants would have given more strength to the study.
- Would have liked to add more questions to the satisfaction with health care section to evaluate parameters that further contribute to knowledge regarding quality of life such as catastrophizing, acceptance, and self-efficacy.
- The data in this study seemed to call for multivariate analysis; however, this was not possible due to the sample size used in the study and study design.

## Figures and Tables

**Table 1:** Demographic data.

***Hospital***	
Hospital 1	57 (63.3%)
Hospital 2	27 (30.0%)
Hospital 3	6 (6.7%)
***Gender***	
Female	48 (53.3%)
Male	42 (46.7%)
***Age (years)***	
1–11	51 (56.7%)
12–18	39 (43.3%)
***School attendance***	
Irregular	50 (83.3%)
Regular	10 (16.7%).
***Blood relation between parents***	
First degree relatives	49 (54.4%)
Second degree relatives	13 (14.4%)
No relation	27 (30.0%)
***Parent education***	
Father primary education	51 (56.7%)
Mother primary education	43 (47.8%)
***Monthly income***	
<500 JD	73 (81.1%)
500–1000 JD	16 (17.8%)
>1500 JD	1 (1.1%)
***Patient’s physical activity***	
Yes	55 (61.1%)
No	35 (38.9%)

Data presented as frequency and (%). JD; Jordanian dinar.

**Table 2:** Disease characteristics.

Characteristic	*n* (%)
***Type of sickle cell disease (SCD)***	
SS Hb	48 (53.3%)
Sβ/Thalassemia	42 (46.7%)
***Reason for admission***	
Feeling pain	4 (4.4%)
Uncontrolled disease	3 (3.3%)
First visit	2 (2.2%)
Follow up of disease	81 (90.0%)
***Surgeries***	
Yes	41 (45.6%)
No	46 (54.4%)
***WBC count***	
High	57 (63.3%)
Normal	32 (35.6%)
***RBC count***	
Low	76 (84.4%)
Normal	14 (15.6%)
***Hg level***	
Low	90 (100%)
Blood transfusion	85 (94.4%)
History of bleeding	5 (5.6%)
***Comorbidity***	
Respiratory disease	26 (28.9%)
Pallor	5 (5.6%)
Urine retention	4 (4.4%)
Tonsillitis	4 (4.4%)
Corrosion in the joint	3 (3.3%)
Stroke	1 (1.1%)
Cystic fibrosis	1 (1.1%)
Swelling of colon	1 (1.1%)

Data presented as frequency and (%); Hg, hemoglobin; RBC, red blood cells; WBC, while blood cells.

**Table 3:** Medications utilized at home and their adverse effects.

Drugs Used Regularly	*n* (%)
Acetaminophen	68 (75.6%)
NSAIDs	67 (74.4%)
Folic acid	88 (97.8%)
Hydroxyurea	51 (65.7%)
Antibiotics	26 (28.9%)
Exjade® (deferasirox)	19 (21.1%)
***Side effects***	
Indigestion	6 (6.7%)
Ulcers in GIT	5 (5.6%)
Diarrhea	4 (4.4%)
Nausea	3 (3.3%)
Headache	2 (2.2%)
Vertigo	1 (1.1%)

Data presented as frequency and (%); NSAIDs: non-steroidal anti-inflammatory drugs; GIT: Gastro-intestinal tract.

**Table 4:** Percentage of patients reporting different words to describe their pain during pain crisis.

Number of Words Used by Patient	Words to Describe Pain		
	Sensory	Affective	Temporal
0	18.9%	14.4%	6.7%
1	51.1%	62.2%	84.4%
2	28.9%	22.2%	8.9%
3	1.1%	1.1%	-

**Table 5:** Correlation analysis between components of the Adolescent Pediatric Pain Tool (APPT) with several variables.

Correlations	Pain Intensity		Number of Body Areas with Pain		Sensory Words		Affective Words		Temporal Words	
	rho	*p*	rho	*p*	rho	*p*	rho	*p*	rho	*p*
Age	**0.519**	**<0.001** **	**0.469**	**<0.001** **	**0.423**	**<0.001** **	0.099	0.369	**0.472**	**<0.001** **
BMI	**0.434**	**<0.001** **	**0.343**	**0.001** **	**0.320**	**0.003** **	0.145	0.184	**0.411**	**<0.001** **
School absences	**0.315**	**0.018** *	**0.405**	**0.002** **	0.215	0.112	0.232	0.085	**0.330**	**0.013** *
Number of surgeries	**0.445**	**<0.001** **	**0.319**	**0.003** **	**0.326**	**0.002** **	−0.071	0.520	0.138	0.208
Pain intensity	-----	-----	**0.649**	**<0.001** **	**0.546**	**<0.001** **	**0.214**	**0.049** *	**0.409**	**<0.001** **
Number of body areas with pain	**0.649**	**<0.001** **	-----	-----	**0.467**	**<0.001** **	**0.337**	**0.002** **	**0.424**	**<0.001** **
Sensory words	**0.546**	**<0.001** **	**0.467**	**<0.001** **	-----	-----	0.119	0.278	**0.328**	**0.002** **
Affective words	**0.214**	**0.049** *	**0.337**	**0.002** **	0.119	0.278	-----	-----	0.086	0.434
Temporal words	**0.409**	**<0.001** **	**0.424**	**<0.001** **	**0.328**	**0.002** **	0.086	0.434	-----	-----

rho: Spearman’s correlation coefficients, *p*: *p*-value, *, Correlation is significant at the 0.05 level; **, Correlation is significant at the 0.01 level; BMI: Body Mass Index. Significant correlations are in bold figures.
